# Trends of changes in physical activity of older adults in Poland 2009–2019. The results of the representative population-based PolSenior2 study

**DOI:** 10.1186/s12889-026-26218-6

**Published:** 2026-01-14

**Authors:** Joanna Kostka, Kacper Jagiełło, Hanna Kujawska‑Danecka, Adam Hajduk, Aleksandra Szybalska, Małgorzata Mossakowska, Tomasz Zdrojewski, Tomasz Kostka

**Affiliations:** 1 Department of Physioprophylaxis, Medical University of Lodz, pl. Hallera 1B, Lodz, 90-647 Poland; 2 Department of Preventive Medicine and Education, Medical University of Gdansk, Gdansk, Poland; 3 Department of Rheumatology, Clinical Immunology, Geriatrics and Internal Medicine, Medical University of Gdansk, Gdansk, Poland; 4 Study on Aging and Longevity, International Institute of Molecular and Cell Biology in Warsaw, 4 Ks. Trojdena Street, Warsaw, 02-109 Poland; 5 Polish Society of Gerontology, Mazovia Branch, Warsaw, Poland; 6 Department of Geriatrics, Medical University of Lodz, Pomorska 247/249, Lodz, 92-209 Poland

**Keywords:** Lifestyle, Elderly, Socio-demographic factors, Central-Eastern europe, PolSenior

## Abstract

**Background:**

Health benefits of regular physical activity (PA) have been widely recognized in older people. The aim of this study was to present the data on leisure time physical activity (LTPA) of Polish citizens aged 65 years and over in the representative population-based PolSenior2 project conducted between 2018 and 2019 and to compare with PolSenior study conducted 10 years earlier.

**Methods:**

The data of the 4,932 participants (2,430 men and 2,502 women) aged over 65 years from the PolSenior2 study with complete PA data were related to the results of the 4,813 participants (2,488 men and 2,325 women) aged 65 and more from the PolSenior study. The methodology was the same in the PolSenior2 as in the PolSenior study.

**Results:**

Gardening (51.5%), walking (35.3%) and cycling (31.2%) were the most popular physical activities, however, with lower prevalence as compared to PolSenior. The percentage of older people engaged in swimming (8.4%), dancing (16.0%) and running or jogging (2.1%)(borderline significance) increased in PolSenior2 as compared to PolSenior study. After summing up the types, intensities and frequencies of different activities as defined in previous PolSenior study, the satisfactory level of LTPA was comparable (*p* = 0.064) in PolSenior2 vs. PolSenior (35.4% vs. 33.6%). Higher level was found for age cohort of 65–69 years, for the residents of villages, for farmers, and for other than blue and white collar workers social class group. In contrast, the LTPA level was lower for the residents of cities with 200,000-500,000 inhabitants in the PolSenior2 as compared to the PolSenior survey.

**Conclusion:**

Health policies should concentrate on a combination of sustaining existing favourable PA habits and developing access and motivation to participate in sports activities characteristic of Western countries.

**Supplementary Information:**

The online version contains supplementary material available at 10.1186/s12889-026-26218-6.

## Introduction

Health benefits of regular physical activity (PA) have been widely recognized in older people [1, 2]. PA in old age is associated with better health status and reduced mortality [3–5]. Older people who engage in regular exercise perform daily activities better and maintain satisfactory mobility [6–9]. PA improves mood and mental well-being, and reduces the risk of cognitive impairment and dementia [7, 10].

Older people constitute the fastest growing segment of the population from Central and Eastern Europe [11, 12]. At early stages of the socioeconomic transformation in Poland, a decline in mortality from coronary heart disease was attributed to changes in major risk factors, mainly reducing total cholesterol concentration and increasing leisure-time PA [13]. Nevertheless, an important East-West health gap is still apparent [14–18]. The prevalence of cardiovascular diseases is still higher in Poland and other Eastern European countries than in Western Europe [19]. A similar situation applies to life expectancy for both women and men. According to the Eurostat report, the highest life expectancy at birth is recorded in Scandinavia and Western European countries and is lower in Eastern European countries, including Poland [20]. Therefore, improving and maintaining a physically active lifestyle is a great challenge for Poland and other countries from Central and Eastern Europe.

There is a constant need of gathering information on the comprehensive PA behaviours and their changes over time in different older populations. According to the available literature, only one large study examined long-term changes of PA from former communist countries [21], and none in selected older population. Therefore, the aim of the present study was to report data on leisure time physical activity (LTPA) of Polish citizens aged 65 years and over from the representative population-based PolSenior2 project and to compare it with PolSenior study conducted 10 years earlier.

## Methods

### Project description

**Fig. 1:**
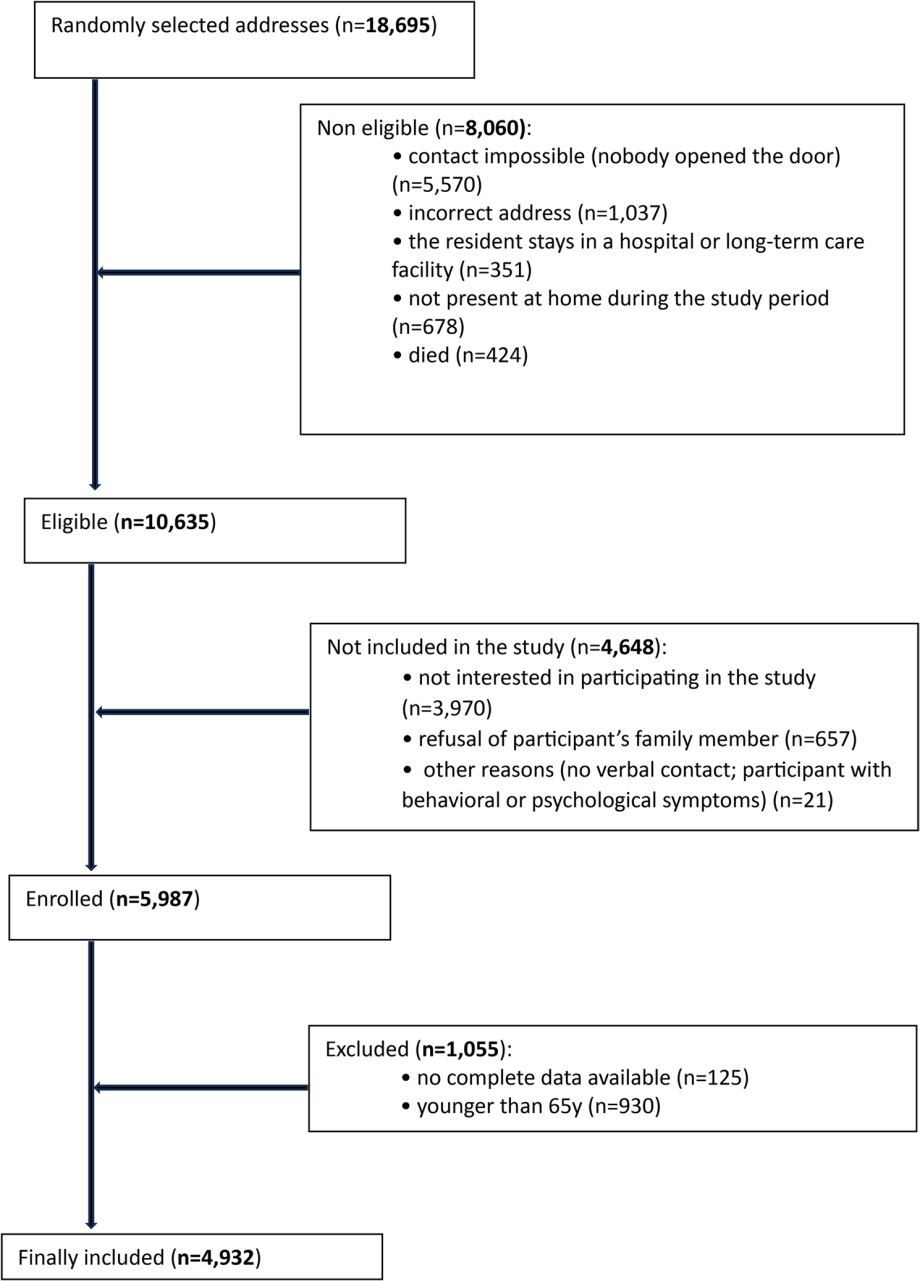
The flow chart of the study

The present study is a part of the PolSenior2 project, a nationwide, multicenter, cross-sectional, face-to-face study of a representative sample of Polish Caucasians, aged over 60 years conducted between 2018 and 2019 in Poland [22]. A random sampling procedure, stratified by age and sex, was employed to select a study group of 5987 Polish community-dwelling adults ≥ 60 years of age, representative of the general population of older Poles, estimated at 8.5 million. A three-stage stratified and proportional sampling was performed: (1) administrative units, (2) random selection of villages in rural municipalities and cities in urban municipalities, and (3) study participants were drawn, based on their PESEL (Universal Electronic System for Registration of the Population) number. The study participants were divided into 5-year age cohorts: 65.0–69.9, 70.0–74.9, 75.0–79.9, 80.0–84.9, 85.0–89.9, and ≥ 90.0 years. There was an attempt to recruit the cohorts similar in number and of similar representation of women and men. Exclusion criteria consisted of inability to establish contact with a selected respondent, hospitalization or institutionalization, other temporary relocations, death prior to the beginning of the study, refusal to participate, and inability to obtain informed consent. Study participants were visited three times by trained medical nurses. During the visits, detailed medical and socioeconomic questionnaires were completed. The details of the recruitment process and study design have been published elsewhere [22]. The present study shows the data of the 4,932 participants (2,430 men and 2,502 women) aged over 65 years with complete PA data. The flow chart of the present sampling procedure has been shown in Fig. 1. The PolSenior2 project was approved by the Bioethics Committee of the Medical University of Gdańsk, Poland (NKBBN/257/2017). Written consent was obtained from all participants prior to enrollment in the study.

Data obtained with the PolSenior2 project were compared to the results of the 4,813 participants (2,488 men and 2,325 women) aged 65 and more from the first PolSenior programme [23]. The PolSenior project was a multicenter, publicly funded research project commissioned by the Ministry of Science and Higher Education, conducted in 2008 and 2009. Detailed methodology of the project has been previously described [24].

### Questionnaire

The study used a questionnaire consisting of a section covering socio-demographic data and a section on PA. The same socio-economic data were collected from all participants of both projects (PolSenior and PolSenior2), including personal and family situation, interests and hobby, economic status, leisure time activities and social life. Data on PA of older Poles were presented in relation to age, sex, size of place of residence, social class and retirement status. The assignment of participants to individual occupational groups (farmer employee, blue-collar employee, white-collar employee and other employee) was based on their answers to the question regarding their employment for the longest period of their lives [24].

Data on PA were obtained based on answers to questions about engaging in PA in free time in the last 12 months. The questions concerned type of PA and frequency of undertaking these activities. The respondents answered the question: ‘‘How often have you engaged in the following activities. Please consider the period of the past 12 months.’’ Possible answers included: once a year, several times a year, once or twice a month, once a week, several times a week, every day, hard to say. All physical activities included in the questionnaire were classified as moderate or vigorous [25]. Moderate intensity activities included in the questionnaire were as follows: (1) walks farther from home or the place of accommodation; (2) gymnastic exercise, aerobics, etc.; (3) riding a bicycle; (4) gardening. Vigorous-intensity activities included in the questionnaire were as follows: (1) running or jogging; (2) swimming; (3) team games (volleyball, basketball, football, etc.); (4) sailing; (5) horse riding; (6) tennis; (7) table tennis; and (8) dancing. To increase the precision of the answers, the questionnaire included examples of various forms of LTPA. A detailed description of the PA part of the questionnaire was presented in a previous publication on the PolSenior project [23].

PolSenior2 data on PA were summed up to compare with the previous PolSenior project. For the purpose of comparison, the same methodology was adopted to the PolSenior2 study to present 10-year trends in LTPA among older Poles. Satisfactory PA level was considered to be met by performing:

- any type of PA every day, or
- high-intensity exercises several times a week, or
- any two forms of moderate intensity exercise several times a week, or
- any three forms of high-intensity exercise once a week, or
- any five forms of moderate intensity exercise once a week, or
- a combination of moderate and vigorous exercise in an amount corresponding to the above criteria, taking into account that one session of intense exercise corresponds to two sessions of moderate intensity.

### Statistical analysis

The data management and the statistical analyses were performed with R version 3.6.3 R (R Core Team, version 3.6.3) and SAS 9.4 TS Level 1M5 (SAS Institute Inc., Cary, NC, USA). Percentages and proportions were compared with chi-square test. Sampling weights were included in statistical calculations to account for the complex survey design using R survey package. The post-stratification procedure was used to match age-sex sample distribution to the population of Poland [26]. The level of significance was set at *p* < 0.05.

## Results

Sociodemographic characteristics of the PolSenior 2 population has been presented in Table 1. This sample is comparable to the PolSenior sample of 4813 older adults assessed for PA in 2008–2009 [23]. Overall, the estimated weighted population prevalence (95% CI) of men was higher in towns with 50,000–200,000 inhabitants, blue-collar workers and retired participants, while that of women was higher in farmers and white-collars groups.

**Table 1:** Sociodemographic characteristics of the PolSenior 2 population according to sex

		Men (*n* = 2430; 100%)	Women (*n* = 2502 100%)	Total (*n* = 4932 100%)	*p*-value for gender difference
Size of place of residence (%)	Village	34.6	35.7	35.2	0.392
	Town up to 20,000	11.9	12.7	12.3	0.359
	Town 20,000–50,000	12.6	13.1	12.9	0.588
	Town 50,000–200,000	20.5	18.2	19.3	0.040
	City 200,000–500,000	12.3	11.5	11.9	0.366
	City ≥ 500,000	8.1	8.8	8.5	0.445
Social class (%)	Farmer	4.8	9.5	7.2	< 0.001
	Blue-collar	60.0	44.0	52.0	< 0.001
	White-collar	25.9	38.4	32.1	< 0.001
	Other	9.3	8.0	8.7	0.120
Retirement status (%)	Retired	94.7	89.6	92.1	< 0.001
	Active	5.3	10.4	7.9	

Chi-square test for sex and size of the place of residence: 0.294

Chi-square test for sex and social class: <0.001

**Fig. 2:**
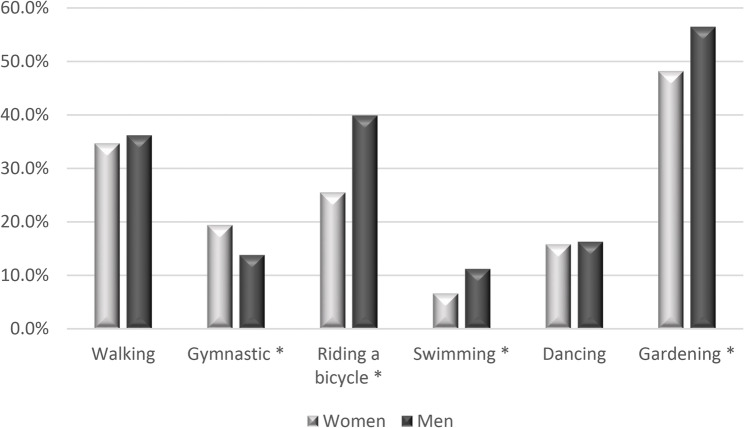
The most popular leisure time physical activities in women and men of the PolSenior2 study (% reporting given activity). * - statistical difference between women and men (chi-square test)

Detailed characteristics of PA of PolSenior2 participants (involvement in particular types and frequency of LTPA have been presented in Appendix Table 1. This table does not include activities that respondents undertook very rarely (tennis, skiing, sailing, horse riding). The most popular LTPA of in women and men of the PolSenior 2 study have been shown in Fig. 2.

Similarly to PolSenior, also in PolSenior 2 the most popular LTPA were gardening, walking and cycling, as well as gymnastic exercises and dancing. More men than women reported riding a bicycle (39.9% vs. 25.5%), swimming (11.2% vs. 6.6%), team games (1.8% vs. 0.7%), table tennis (1.7% vs. 0.1%) and gardening (56.5% vs. 48.2%)(chi-square test). There were no sex-related differences in reported frequency of farther walking (36.2% vs. 34.7%), running or jogging (2.5% vs. 1.9%) and dancing (16.3% vs. 15.8%). More women than men reported gymnastics (19.4% vs. 13.8%). For the majority of activities, we observed that the older age groups were less active. Frequency of reported table tennis was relatively stable with advancing age. Village residents reported farther walking, gymnastics, running and swimming (31.1%, 12.0%, 1.0%, 5.6%, respectively) less frequently than those who live in cities, especially as compared to the residents of cities of 500,000 inhabitants and greater (45.5%, 23.9%, 4.1%, 14.9%, respectively). In contrast, gardening generally decreased with the size of the place of residence. Village residents reported gardening more frequently (62.0%) than the residents of the largest cities (40.8%). Similar differences were found when respondents were filtered by social and professional group, with the most statistically significant differences observed between farmers and white-collar workers. Farmers reported farther walking, gymnastics, running or jogging, swimming and dancing (21.0%, 2.8%, 0.2%, 1.2%, 9.1% respectively) less frequently than other social classes, especially as compared to white-collar workers (44.6%, 29.2%, 3.1%, 13.9%, 23.4%, respectively) (Appendix Table 1). Riding a bicycle and gardening was more often reported by retired seniors, other forms of PA were not related to the retirement status (not shown in the Table).

Comparison of LTPA of respondents in PolSenior and PolSenior2 has been presented in Table 2. The percentage of older adults engaged in walks farther from home or the place of accommodation, riding a bicycle and gardening decreased in 2018–2019 as compared to 2008–2009. In contrast, the percentage of older adults engaged in swimming, dancing and running or jogging (borderline significance) increased in PolSenior2 as compared to PolSenior study.

**Table 2:** Comparison of leisure time physical activities of respondents in PolSenior and PolSenior2

	PolSenior Percentage of older adults engaged in the following activities in their free time	PolSenior2 Percentage of older adults engaged in the following activities in their free time	Difference (Chi^2^) *p*-value
Walks farther from home or the place of accommodation	39.0%	35.3%	< 0.001
Gymnastic exercise, aerobics, etc.	18.2%	17.2%	0.193
Riding a bicycle	37.3%	31.2%	< 0.001
Running or jogging	1.6%	2.1%	0.063
Swimming	6.3%	8.4%	< 0.001
Team games (volleyball, basketball, football, etc.)	0.8%	1.1%	0.119
Table tennis	0.9%	0.7%	0.255
Dancing	12.8%	16.0%	< 0.001
Gardening	64.6%	51.5%	< 0.001

Comparison of achieving the satisfactory level of PA as defined in the previous Polsenior study was presented with reference to sex, age, size of the place of residence and social class. These data have been shown in Table 3. In both studies compliance was higher in men and lower in older age. In the PolSenior study, the residents of cities with 500,000 inhabitants or more were characterized by lower compliance in comparison with the residents of villages and the smallest towns. In the PolSenior2 study, the residents of towns with 50,000–200,000 inhabitants were characterized by lower compliance in comparison with the residents of villages and the smallest towns. In the PolSenior2 study, the residents of cities with 200,000–500,000 inhabitants were characterized by lower compliance in comparison with the residents of villages, the smallest towns, and the towns with 50,000–200,000 inhabitants. In the PolSenior study, farmers had lower compliance than other social classes. In the PolSenior2 study, the compliance was not statistically different between the social class groups. Overall satisfactory level of LTPA was slightly higher (*p* = 0.064) in PolSenior2 vs. PolSenior. Higher compliance was found for age cohort of 65–69 years, for the residents of villages, for farmers, and for other than blue and white collar workers social class group. In contrast, the LTPA level was lower for the residents of cities with 200,000–500,000 inhabitants in the PolSenior2 as compared to the PolSenior survey.

**Table 3:** Comparison of achieving the satisfactory level of PA as defined in the previous PolSenior study

		PolSenior Compliance (%)	PolSenior2 Compliance (%)	Polsenior2 vs. PolSenior difference Chi^2^ *p*-value
Total		33.6%	35.4%	0.064
Sex	Men	40.9%	40.2%	0.612
	Women	29.2% *	32.3% *	0.258
Age cohort	65–69 y.o.	41.4%	45.1%	0.037
	70–74 y.o.	37.8%	40.4%	0.180
	75–79 y.o.	31.9% †	34.5% †	0.240
	80–84 y.o.	20.2% † ‡ §	20.7% †‡§	0.838
	85–89 y.o.	12.2% † ‡ §	15.3% † ‡§	0.307
	≥ 90 y.o.	6.9% † ‡ § ||	6.3% †‡§||	0.953
Size of the place of residence	Village	35.4%	40.9%	< 0.001
	Town up to 20,000	41.3%	43.3%	0.508
	Town 20,000–50,000	33.9%	35.6%	0.572
	Town 50,000–200,000	32.6%	28.7% ¶ #	0.114
	City 200,000–500,000	31.4%	24.1% ¶ # ^	0.023
	City ≥ 500,000	27.2% ¶ #	28.1%	0.675
Social class	Farmer	25.3%	32.7%	0.013
	Blue collar	33.6% **	34.7%	0.408
	White collar	37.8% **	37.9%	0.950
	Other	33.2% **	39.3%	0.052

* different to men group

† different to 65–69 year group

‡ different to 70–74 year group

§ different to 75–79 year group

|| different to 80–84 year group

¶ different to village group

# different to town up to 20,000 group

^ different to Town 20,000–50,000 group

** different to farmer group

## Discussion

This is the first representative study portraying 10-year changes in LTPA of an older population in Central-Eastern Europe. Obtained data show several important trends in LTPA behaviours approaching more Western-European pattern of LTPA. Nevertheless, as reported in the 2022 European Commission Special Eurobarometer, several differences with the most active Western societies are still evident with Poland being among the least active nations [27].

According to the results of the present study approximately one third of the Polish older population presents satisfactory PA level. These data are generally comparable to some other studies with older adults from Poland. In a questionnaire sample of 2,023 Poles aged ≥ 60 years, more than 20% participants were completely inactive and almost 40% did not follow the WHO recommendations regarding PA [28]. In a cross-sectional study in 858 older people living in south-eastern Poland, only 25.64% performed a minimum of 150 min of moderate-intensity exercise, while strengthening exercises were performed by 22.49% [29]. In the National Test for Poles’ Health online study of Internet users, over 65% of older (65 years old) respondents claimed to engage in less than 1 h of PA per week [30].

Overall fulfilment of the satisfactory PA level was comparable in PolSenior2 vs. PolSenior (35.4% vs. 33.6%). Nevertheless, this small change (1.8%) may be considered as generally positive, especially that data from younger populations of Poland and other rapidly developing countries indicate rather negative trends in PA behaviours. In one available prospective study that comprised two independent samples of randomly selected adults aged 20–74 years in Poland, unfavorable (associated with a decrease in PA levels) 10-year changes in PA were observed [21]. PA was assessed in three domains: leisure-time, occupational and commuting PA. The prevalence of people being active on most days of week fell in both sexes in the years 2003–2014 (37.4% vs. 27.3% in men; 32.7% vs. 28.3% in women). In both surveys, the likelihood of physical inactivity was higher in less educated individuals, smokers and those living in large agglomerations. Commuting PA decreased significantly in both sexes with an increase in “passive” commuting which also contributed to insufficient PA [21]. Based on leisure time and transport activities, the prevalence of adults aged ≥ 15 years in Poland reaching the recommended PA levels in 2014 was 23% in males and 18% in females [31]. In the European Union (EU), the percentage of respondents who say they never exercise or play sport has gained 6% points from 2009 to 2022, up from 39% to 45% [27]. The prevalence of sedentary behaviors increased between 2002 and 2017 for the EU as a whole and for both sexes separately [32]. Trend analyses show similar declines of occupational PA and domestic PA in other developed or rapidly developing countries [33, 34].

The prevalence of insufficient PA in 65–75-year-olds varies widely between European countries, ranging from 55.4% to 83.3% in women and from 46.6% to 73.7% in men [35]. In the study assessing sedentary behaviors and PA in four European countries, older adults spent 78.8% of waking time in sedentary behaviors, 18.6% in light-intensity PA, and 2.6% in moderate-to-vigorous PA [36]. As much as 62.2% participants of DO-HEALTH study (mean age 74.9 years, 61.8% women) met WHO PA recommendations [37]. Although better than in younger age groups, data on PA behaviours in older Poles are still far from satisfactory. In the cross-sectional analysis with data from participants aged 55 or older in Wave 4 of the Survey of Health, Ageing, and Retirement in Europe (SHARE) database, the overall prevalence of inactivity among individuals age 55 or older in the 16 included countries was 12.5% [38]. The prevalence of physical inactivity varied between countries, ranging from 4.9% (Sweden) to 25.8% (Poland) and 29% (Portugal). Generally, Eastern and Southern European countries were less active [38]. In the other study of older European citizens aged 65 and over, 55.5% of participants were adequately active, and 43.8% were highly active, especially in the North and West [39]. The results from Eurobarometer show that 65.3% of older EU citizens over the age of 60 engage in some form of LTPA, that 40.4% do so for health reasons, and that only 32.3% engage in LTPA that meets the minimum guidelines set by the WHO. People in the countries of the Nordic model of social welfare were the most active [40, 41].

Age, sex and health status have usually been found to be the most important factors influencing participation in PA [29, 36–38]. E.g., in Czech adults, the level of PA decreased with age, men were generally more physically active than women [42]. Socioeconomic features, facility access, neighbourhood characteristics and meteorological factors have also been reported to influence the engagement of older people in a physically active lifestyle [43–46]. Higher level of education is generally associated with achieving the guideline amount of PA [47–49]. The lower the education level and the smaller the place of residence, the greater the inactivity [28]. Changes in movement behavior during the retirement transition are potentially more favorable for high socioeconomic status adults [50]. Socioeconomic factors such as low educational level and financial difficulties, and health-related factors such as number of chronic diseases were associated with insufficient PA in the SHARE study [35]. Among older people in the EU (Eurobarometer), men, people with high socio-economic status, belonging to the middle and upper social classes, living in rural areas where there is infrastructure for PA, and above all, in the Nordic countries, were the most active [40]. However, in the cross-sectional data of 1507 participants (52.5% female) of the OUTDOOR ACTIVE study between 65 and 75 years, residing in Germany, time spent on housework, gardening, biking, and walking decreased with increasing socioeconomic status [49].

As in previous studies, in PolSenior2 age was a powerful factor decreasing compliance to the satisfactory PA level. Of interest is an increased compliance of the youngest seniors (65–69 y.o.) in PolSenior2 vs. PolSenior study. Higher level of education tended to be associated with better compliance, however, this relationship was not as evident in the PolSenior2 as it was in the PolSenior study. Interestingly, compliance clearly improved in “farmers” and “other” social classes during 10 years. It increased in village residence but decreased in middle-size cities. The interpretation of this trends includes white-collars workers moving their place of residence outside cities to suburbs and villages, and increasing education level of farmers group. Overall, it seems that multiple healthy lifestyle-promoting campaigns, usually stressing importance of PA in older adults, give beneficial results in this age group, contrasting with PA changes in younger populations.

In the present study, gardening (51.5%), walking (35.3%) and cycling (31.2%) were the most popular physical activities, however with lower prevalence as compared to PolSenior. Gardening, as a part of non-intentional exercise (or non-exercise PA), may be considered as an important health-promoting behaviour in the older population [51–55]. Gardening alleviates muscle mass and functional decline, reduces falls incidence, improves cardiovascular health and promotes longevity in older population [56–61]. In Poland, family allotments originate from the socialist period, when they were allowed to the “working classes” by the authorities. They are usually located near residencies, within or close to cities. Two million of Poles, usually older people, are members of allotment organizations. This characteristic feature of community gardening in Poland (and probably other post-communist European countries) should merit special attention by public health authorities [62, 63]. Especially, that due to growing land prices allotment gardens situated within large cities are in danger of liquidation.

Walking is the main LTPA of older European citizens [39, 64]. In the US nationally representative sample major activities among active older adults were walking (men, 69%; women, 75%) and gardening (men, 45%; women, 35%) [65]. Walking, gardening and home exercises were the three most frequent types of reported physical activities in older Canadians [47].

Cycling acts as both a form of PA and a mode of transportation. More than 30% of PolSenior2 respondents reported riding a bicycle, men more often than women, and the frequency of cycling decreased with older age. Interestingly, unlike in PolSenior study, the frequency of cycling was not dependent on the size of the residence or social class, what may reflect the ongoing changes in lifestyle of Polish older adults - decreasing role of cycling as a means of transport among the rural population and farmers.

Our research is a representative population-based study, but it may have some weaknesses. Both studies were planned to ascertain their representativeness but exact statistical identity was not ascertained. The data obtained in the study were based on a questionnaire in which the level of PA was declared by older adults. There is a possibility that the information provided by the respondents may have been over-reporting. On the other hand, the study only took into account leisure-time activity, and did not assess PA related to professional work or household duties. Therefore, especially in the group of women, the total PA level may have been underestimated. Moreover, especially in a rural environment, gardening could be not only an element of free-time activity, but also the basis of professional work related to food production. The exact duration of different activities was not registered. The duration of many activities is highly variable (e.g. gardening, bicycling) and it is impossible in large epidemiological studies to recollect by older adults exact duration of each activity from preceding year. Nevertheless, the PA assessment methodology in the Polsenior and PolSenior2 studies was exactly the same, which means that it is possible to draw conclusions regarding trends in PA over 10 years of observation. Finally, with multiple analyses the chance of committing a type I (false positive results) error increases. On the other hand, correcting for multiple comparisons increases the chance of type II errors (false negative results) occurs, and this adjustment makes finding genuine effects extremely unlikely, even in large studies. Therefore, we decided not to correct for multiple comparisons in the present study, as in previous studies from both PolSenior and PolSenior2. Nevertheless, a possibility of committing a type I error with such an approach should be acknowledged.

## Conclusions

Several important trends in PA of Polish older population have emerged. Decreasing frequency of gardening, cycling and walking may be considered as unfavourable changes related to the modernisation of transport and diminishing habitual village population with their traditional lifestyle. On the other hand, more often reported “western” types of PA like swimming should be assessed positively. Therefore, though not entirely satisfactory, the overall trend of changes in LTPA level of Polish older adults seems to be more favourable than that of younger adults. Health policies should concentrate on a combination of sustaining existing favourable PA habits and developing access and motivation to participate in sports activities characteristic of Western countries. By enforcing favourable changes and taking advantage of existing behaviours, the East-West health gap in LTPA of a former communist country may be further reduced, more than thirty years after the beginning of the socio-economic transition.

## Supplementary Information

Supplementary Material 1.

## Data Availability

The study is a part of the nationwide PolSenior2 project, and the data have been deposited in the Polish Ministry of Health.
