# Supplementary material for: Trends of changes in physical activity of older adults in Poland 2009–2019. The results of the representative population-based PolSenior2 study
Source: BMC Public Health. 2026 Jan 14;26:550. doi: 10.1186/s12889-026-26218-6 (PMC12888410; doi:10.1186/s12889-026-26218-6)
Supplement: Supplementary file 1 — Supplementary Material 1. [file 12889_2026_26218_MOESM1_ESM.doc]

Appendix Table 1. Leisure time physical activities of respondents of the PolSenior 2 population.

|  | Have you engaged in the following activities in your free time? | For “yes” answer (yes = 100%)  How often have you spent your time in this way? | | | | | | |
| --- | --- | --- | --- | --- | --- | --- | --- | --- |
|  | Yes | once a year | several times a year | once or twice a month | once a week | several times a week | every day | hard to say |
| Walks farther from home or the place of accommodation | 35.3 (32.9-37.7) †‡§ | 2.0 (1.0-3.0) | 16.1 (13.3-18.9) | 24.6 (21.1-28.1) | 20.1 (17.4-22.7) | 26.1 (22.4-29.9) | 10.9 (8.7-13.2) | 0.2 (0-0.5) |
| Gymnastic exercise, aerobics, etc. | 17.2 (15.3-19.0) *†‡§ | 0.2 (0-0.4) | 4.5 (2.5-6.4) | 13.7 (9.0-18.5) | 18.5 (14.5-22.5) | 28.1 (22.5-33.6) | 34.9 (30.0-39.9) | 0.1 (0-0.3) †‡§ |
| Riding a bicycle | 31.2 (28.6-33.8) *† | 0.3 (0-0.6) | 8.5 (5.8-11.2) | 16.6 (13.3-19.9) | 13.9 (11.1-16.8) | 30.6 (26.6-34.6) | 29.5 (25.7-33.3) | 0.5 (0.1-0.9) † |
| Running or jogging | 2.1 (1.5-2.7)  †‡§ | 2.1 (0-5.0) | 16.1 (6.6-25.6) | 13.2 (3.6-22.8) | 23.7 (14.9-32.6) | 23.2 (11.4-34.9) | 18.3 (4.6-32.1) | 3.3 (0-7.4) *†§ |
| Swimming | 8.4 (7.1-9.7)  *†‡§ | 5.1 (2.1-8.1) | 41.5 (34.8-48.2) | 23.9 (16.8-31.0) | 17.4 (12.8-21.9) | 7.8 (2.8-12.8) | 4.0 (0.5-7.5) | 0.3 (0-0.8) *† |
| Team games (volleyball, basketball, football, etc.) | 1.1 (0.7-1.5)  *† | 5.4 (0.2-10.5) | 47.8 (28.7-67.0) | 21.9 (3.8-40.0) | 14.2 (3.5-24.8) | 9.8 (0-22.1) | 1.0 (0-2.8) | 0 (0-0)  †‡§ |
| Table tennis | 0.7 (0.4-1.0)  * | 5.9 (0-13.0) | 62.3 (45.5-79.2) | 19 (4.7-33.4) | 2.1 (0-6.1) | 8.7 (0-20.9) | 1.6 (0-4.8) | 0.3 (0-0.9) *†‡§ |
| Dancing | 16.0 (14.0-18.0) †§ | 18.4 (14.1-22.6) | 58.0 (50.7-65.3) | 11 (7.8-14.1) | 4.8 (2.5-7.2) | 3.3 (0.9-5.6) | 4.1 (0.6-7.6) | 0.4 (0-1)  * |
| Gardening | 51.5 (48.9-54.1) *†‡ | 0.5 (0.1-0.9) | 6.9 (5.2-8.5) | 10.1 (8.1-12) | 13.2 (10.8-15.7) | 34.0 (30.2-37.9) | 34.7 (30.3-39.0) | 0.7 (0.3-1.1) ‡ |

Data are presented as % (95% confidence interval)

* significant (p<0.05) chi-square statistics (“no” vs. “yes” answer or distribution of “yes” answers) for sex

† significant (p<0.05) chi-square statistics (“no” vs. “yes” answer or distribution of “yes” answers) for age cohort

‡ significant (p<0.05) chi-square statistics (“no” vs. “yes” answer or distribution of “yes” answers) for size of the place of residence

§ significant (p<0.05) chi-square statistics (“no” vs. “yes” answer or distribution of “yes” answers) for social class
